# Supplementary material for: Functional and Genetic Characterization of Porcine Beige Adipocytes
Source: Cells. 2022 Feb 21;11(4):751. doi: 10.3390/cells11040751 (PMC8870396; doi:10.3390/cells11040751)
Supplement: Supplementary file 1 [file cells-11-00751-s001.zip › Supplementary table s3.pdf]

Table S3. The primers for qPCR

Name	Sequence
UCP3	F: CAACAGGAAGTACAGCGGGA<br>R: GTGATGTTGGGCAGAATTCCTTT
PDK4	F: GGCAGCAGTGGTCCAAGAT<br>R: CGACTGTAGCCCTCATTGCA
CIDEA	F: GTCAAGGCCACCATGTACGA<br>R: AGCATTCGGAGCATGTACGT
EBF2	F: AACAACGGCACCCACTACAA<br>R: TATGGGCTGCTTGGTGACTG
DIO2	F: CCTCTTCCTGGCGCTCTATG<br>R: GTAGGCATCGAGGAGGAAGC
CD137	F: ACTGTGCTGCTGGTCATGAA<br>R: AACACCTTCACACTTCCTGCA
ADCY5	F: CAGACATCAACGCCAAGCAG<br>R: AAGCCCTCGATGTCAGCAAA
LEPTIN	F: TGGCCCTATCTGTCCTACGT<br>R: TGGACAGACTCAGGACAGGA
TCF21	F: AGGCTCAAGACCACACTGC<br>R: TCTTTCAGGTCACTTTCGGGTT
ADIPOQ	F: AGATTGGCTGGTTGATGCTCA<br>R: AAACCAGATGTCACACTTGCC
FABP4	F: GGCCAAACCCAACCTGATCA<br>R: CATCCCACTTCTGCACCTGT
PPARG	F: GTCATGGGTGAAACTCTGGGA<br>R: TGTCAACCATGGTCACCTCTTG
ITGA2	F: GGCCTCCCAGAAGCAAAGAT<br>R: TCTCCCATTCGGTTCTCAGGA
HMGA1	F: GGAAAAGGACGGCACTGAGA<br>R: CTTCTGACTCCCTACCAGCG
HMGA2	F: AAGAGTCCCTCCAAAGCAGC<br>R: CACCCATTTCCTAGGTCTGCC
HMGB1	F: TTTGAAGACATGGCAAAGGCG<br>R: AAAACAAGAAAAAGGCCGAAGGAG
HMGB2	F: CAAGGGTGACAAGAAGGGCA<br>R: CTTTGGGCGATGTTCAGAGC
BMP2	F: CCTACATGCTGGACCTGTACC<br>R: TTAAAGAAGAAGCGCCGGGT

CTCF	F: TAGTGGAGAATTGGTGCGGC<br>R: GTGTCCCTGCTGGCATAACT
AMCF-II	F: AGGCGGCTGTAGTGAGAGA<br>R: CCATTCTTCAGGGTGGCTATCA
KIF18A	F: CTGCTGAACCTGGAGTGATGTA<br>R: ACCCTTGAACGACTACCCCT
ANKZF1	F: CAGCGATGATAATGAGGGGCT<br>R: AGGTCTCGTTTCCTCTCCCT
SEMA3A	F: CAGAAGGGATGAATGCAAGTGG<br>R: GCAAATTGGATGAAAAGCCCCA
CNN1	F: AGCCCCACGACATCTTTGAA<br>R: CCACGTTCACCTTGTTCCCT
IGFBP5	F: GTTTGCCTCAACGAAAAGAGCT<br>R: TCAGCTTCTTTCTGCGGTCC
COL1A1	F: CATGACCGAGACGTGTGGAA<br>R: GGACAGTTCTTGATTTCGTCGC
RIMKLB	F: GGACATTTCAAGAGTTGGCAGG<br>R: CACGTACATCCCTTCCATGAGA
COL11A1	F: AACTCAGTGCCCCAACGAAA<br>R: ATCGCCCAACCTCAATACCG
STC2	F: GCAGAACACAGCGGAAATCC<br>R: TCCCATGTAAGCCCCGAATC
HSPB6	F: TGAGCACGGATACATCGCG<br>R: GATGGACAGGACGCCTTCAG
ZNF423	F: GCGCTCGGTGAAAGTTGAAG<br>R: TCACGCTGTTCCTGTCTTCC
ADGRD1	F: GAACATTGGCATCCTCATCGC<br>R: CAGCAAGCACACCAAAGAGC
18S	F: GTAACCCGTTGAACCCCATT<br>R: CCATCCAATCGGTAGTAGCG
